# Supplementary material for: HIVprotI: an integrated web based platform for prediction and design of HIV proteins inhibitors
Source: J Cheminform. 2018 Mar 9;10:12. doi: 10.1186/s13321-018-0266-y (PMC5845081; doi:10.1186/s13321-018-0266-y)
Supplement: Supplementary file 2 — Additional file 2. Source code of HIVProtI web server. [file 13321_2018_266_MOESM2_ESM.zip › HIVprotI_Source-code/hivprotI/weka-3-6-6/doc/weka/core/matrix/CholeskyDecomposition.html]

CholeskyDecomposition


---


|  |  |  |
| --- | --- | --- |
| **Overview** \| **Package** \| **Class** \| **Tree** \| **Deprecated** \| **Index** \| **Help** \| **Weka's home** | |  |
| PREV CLASS   **NEXT CLASS** | **FRAMES**    **NO FRAMES**     **All Classes** |  |
| SUMMARY: NESTED \| FIELD \| CONSTR \| METHOD | DETAIL: FIELD \| CONSTR \| METHOD |  |


---


## weka.core.matrix Class CholeskyDecomposition

```
java.lang.Object
  weka.core.matrix.CholeskyDecomposition
```

**All Implemented Interfaces:**: java.io.Serializable, RevisionHandler

---

``` public class CholeskyDecomposition extends java.lang.Object implements java.io.Serializable, RevisionHandler ```

Cholesky Decomposition.

For a symmetric, positive definite matrix A, the Cholesky decomposition is
an lower triangular matrix L so that A = L\*L'.

If the matrix is not symmetric or positive definite, the constructor
returns a partial decomposition and sets an internal flag that may
be queried by the isSPD() method.

Adapted from the JAMA package.

**Version:**
:   $Revision: 1.5 $

**Author:**
:   The Mathworks and NIST, Fracpete (fracpete at waikato dot ac dot nz)

**See Also:**: Serialized Form

---

**Constructor Summary**

`CholeskyDecomposition(Matrix Arg)`
  
          Cholesky algorithm for symmetric and positive definite matrix.


| **Method Summary** | |
| --- | --- |
| `Matrix` | `getL()`             Return triangular factor. |
| `java.lang.String` | `getRevision()`             Returns the revision string. |
| `boolean` | `isSPD()`             Is the matrix symmetric and positive definite? |
| `Matrix` | `solve(Matrix B)`             Solve A\*X = B |

**Methods inherited from class java.lang.Object**

`equals, getClass, hashCode, notify, notifyAll, toString, wait, wait, wait`

**Constructor Detail**

### CholeskyDecomposition

```
public CholeskyDecomposition(Matrix Arg)
```

:   Cholesky algorithm for symmetric and positive definite matrix.

    **Parameters:**: `Arg` - Square, symmetric matrix.


**Method Detail**

### isSPD

```
public boolean isSPD()
```

:   Is the matrix symmetric and positive definite?

    :   **Returns:**: true if A is symmetric and positive definite.

---


### getL

```
public Matrix getL()
```

:   Return triangular factor.

    :   **Returns:**: L

---


### solve

```
public Matrix solve(Matrix B)
```

:   Solve A\*X = B

    :   **Parameters:**: `B` - A Matrix with as many rows as A and any number of columns. **Returns:**: X so that L\*L'\*X = B **Throws:**: `java.lang.IllegalArgumentException` - Matrix row dimensions must agree.: `java.lang.RuntimeException` - Matrix is not symmetric positive definite.

---


### getRevision

```
public java.lang.String getRevision()
```

:   Returns the revision string.

    :   **Specified by:**: `getRevision` in interface `RevisionHandler`
    :   **Returns:**: the revision


---


|  |  |  |
| --- | --- | --- |
| **Overview** \| **Package** \| **Class** \| **Tree** \| **Deprecated** \| **Index** \| **Help** \| **Weka's home** | |  |
| PREV CLASS   **NEXT CLASS** | **FRAMES**    **NO FRAMES**     **All Classes** |  |
| SUMMARY: NESTED \| FIELD \| CONSTR \| METHOD | DETAIL: FIELD \| CONSTR \| METHOD |  |


---
